# Transcriptome changes induced by Arbuscular mycorrhizal symbiosis in leaves of durum wheat (*Triticum durum* Desf.) promote higher salt tolerance

**DOI:** 10.1038/s41598-022-26903-7

**Published:** 2023-01-03

**Authors:** Guglielmo Puccio, Rosolino Ingraffia, Francesco Mercati, Gaetano Amato, Dario Giambalvo, Federico Martinelli, Francesco Sunseri, Alfonso S. Frenda

**Affiliations:** 1 Department of Agricultural, Food and Forestry Sciences, University of Palermo, Palermo, Italy; 2 Plant Ecology, Institute of Biology, Freie Universität Berlin, Berlin, Germany; 3 Berlin-Brandenburg Institute of Advanced Biodiversity Research, Berlin, Germany; 4 Institute of Biosciences and BioResources (IBBR), National Research Council of Italy, Palermo, Italy; 5 Department of Biology, University of Florence, Sesto Fiorentino, Italy; 6 Department of Agraria, University Mediterranea of Reggio Calabria, Reggio Calabria, Italy

**Keywords:** Plant genetics, Plant genetics, Plant stress responses, Plant symbiosis, Arbuscular mycorrhiza, Bioinformatics

## Abstract

The salinity of soil is a relevant environmental problem around the world, with climate change raising its relevance, particularly in arid and semiarid areas. Arbuscular Mycorrhizal Fungi (AMF) positively affect plant growth and health by mitigating biotic and abiotic stresses, including salt stress. The mechanisms through which these benefits manifest are, however, still unclear. This work aimed to identify key genes involved in the response to salt stress induced by AMF using RNA-Seq analysis on durum wheat (*Triticum turgidum* L. subsp. *durum* Desf. Husn.). Five hundred sixty-three differentially expressed genes (DEGs), many of which involved in pathways related to plant stress responses, were identified. The expression of genes involved in trehalose metabolism, RNA processing, vesicle trafficking, cell wall organization, and signal transduction was significantly enhanced by the AMF symbiosis. A downregulation of genes involved in both enzymatic and non-enzymatic oxidative stress responses as well as amino acids, lipids, and carbohydrates metabolisms was also detected, suggesting a lower oxidative stress condition in the AMF inoculated plants. Interestingly, many transcription factor families, including WRKY, NAC, and MYB, already known for their key role in plant abiotic stress response, were found differentially expressed between treatments. This study provides valuable insights on AMF-induced gene expression modulation and the beneficial effects of plant-AMF interaction in durum wheat under salt stress.

## Introduction

Soil salinity is one of the most severe environmental stresses in agricultural systems due to its adverse effects on crop growth and productivity^1^. Approximately 20% of cultivated and almost 30% of irrigated lands are affected by soil salinization^2^. High salt deposition in soil affects plant development by inducing physiological, biochemical, and molecular modifications, resulting in cellular osmotic stress, and ionic and redox imbalances. Plants evolved several mechanisms to avoid damage due to salt stress, mainly by ion-homeostasis, solute accumulation, water and nutrient uptake regulation, and ROS detoxification through antioxidant enzymes and molecules^3–5^. The interactions between plants and microorganisms (Plant-Growth-Promoting microorganisms and Arbuscular Mycorrhizal Fungi—AMF) are also reported to play a key role in the crop yield maintenance under stress environments^6,7^.

More than 80% of land plants, including several crop species, are symbiotically associated with AMF^8^. This symbiosis positively impacts plant health and the ability to mitigate both biotic and abiotic stresses through several mechanisms, such as root architecture change, higher accumulation of osmolytes, enhancement of the antioxidant defense system, maintenance of ion homeostasis, and higher photosynthetic efficiency^9–14^. The plant ability to explore a higher soil volume due to the fungal hyphae results in a higher water and nutrient (P, N, Mg, and Ca) uptake by mycorrhized plants^15–17^. These positive effects could be attributable to an increased expression of many genes involved in plant nutrition in AMF colonized plants. Indeed, genes related to proline, trehalose, soluble sugars, and other osmolytes are often upregulated in mycorrhized plants to contrast cell dehydration caused by the lower turgor^18,19^. In addition, AMF symbiosis is also responsible for increased enzymatic and non-enzymatic oxidative stress responses by the upregulation of peroxidases (POX), catalases (CAT), and superoxide dismutase (SOD) and by incresing the synthesis of antioxidant compounds^20^. A Na^+^ and Cl^-^ high concentration in the soil can also lead to harmful ionic imbalances. AMF were reported to enhance ion homeostasis by regulating organic acids and polyamines concentration as well as enhancing Na^+^/K^+^ and Na^+^/H^+^ transporters (HKT, NHX and SOS) expression, to support Na^+^ efflux from cytosol to apoplast or into the vacuole^21^. Furthermore, nitrogen (N) plays a crucial role in mitigating the salt stress^22,23^. Soil salinity can stimulate the expression of NO_3_^−^ transporters and increase NR activity, according to many gene expression analyses^24–26^. Indeed, the accumulation of N-containing metabolites is an important strategy to mitigate both osmotic and oxidative stress^27^. Interestingly, these processes are often enhanced by AMF root colonization resulting in the higher expression of plant NRT transporters and proton ATPases^28^. All these mechanisms help mycorrhized plants to cope with soil salinity resulting in improved plant growth compared to the uninoculated plants.

Although many efforts have been made in recent years to improve knowledge of the complex mechanisms underlying salt stress response in model plants^29,30^, few studies have focused on transcriptional regulation in non-model plants, including durum wheat. The agronomic, metabolic and proteomic responses to salt stress in durum and bread wheat have been described^31,32^, while the transcriptional changesinduced by salinity were reported mainly in the bread wheat (*Triticum aestivum*) roots^33–35^. Targeted comparative analysis of transcripts abundance by qPCR on durum wheat under salt stress and its mitigation effect by AMF symbiosis have ben reported^26,36^. Interestingly, a positive effect of AMF symbiosis on plant biomass, N uptake, and plasma membranes stability in durum wheat under salt stress were detected^26^, highlighting also a significant drought-related genes downregulation in the AMF-inoculated plants.

A wide comparative transcriptomic analysis between AMF inoculated and uninoculated durum wheat plants grown under salt stress will help to better understand the mechanisms involved in salinity stress tolerance mediated by AMF symbiosis.

Thus, after assessing the effects of mycorrhization on productive and qualitative traits of durum wheat grown under salt stress, the putative key genes involved in the improved response of mycorrhized plants to salt stress were identified using RNA-seq analysis.

## Results

### Effects of arbuscular mycorrhizal fungi on durum wheat plants grown under salinity stress

**Figure 1:**
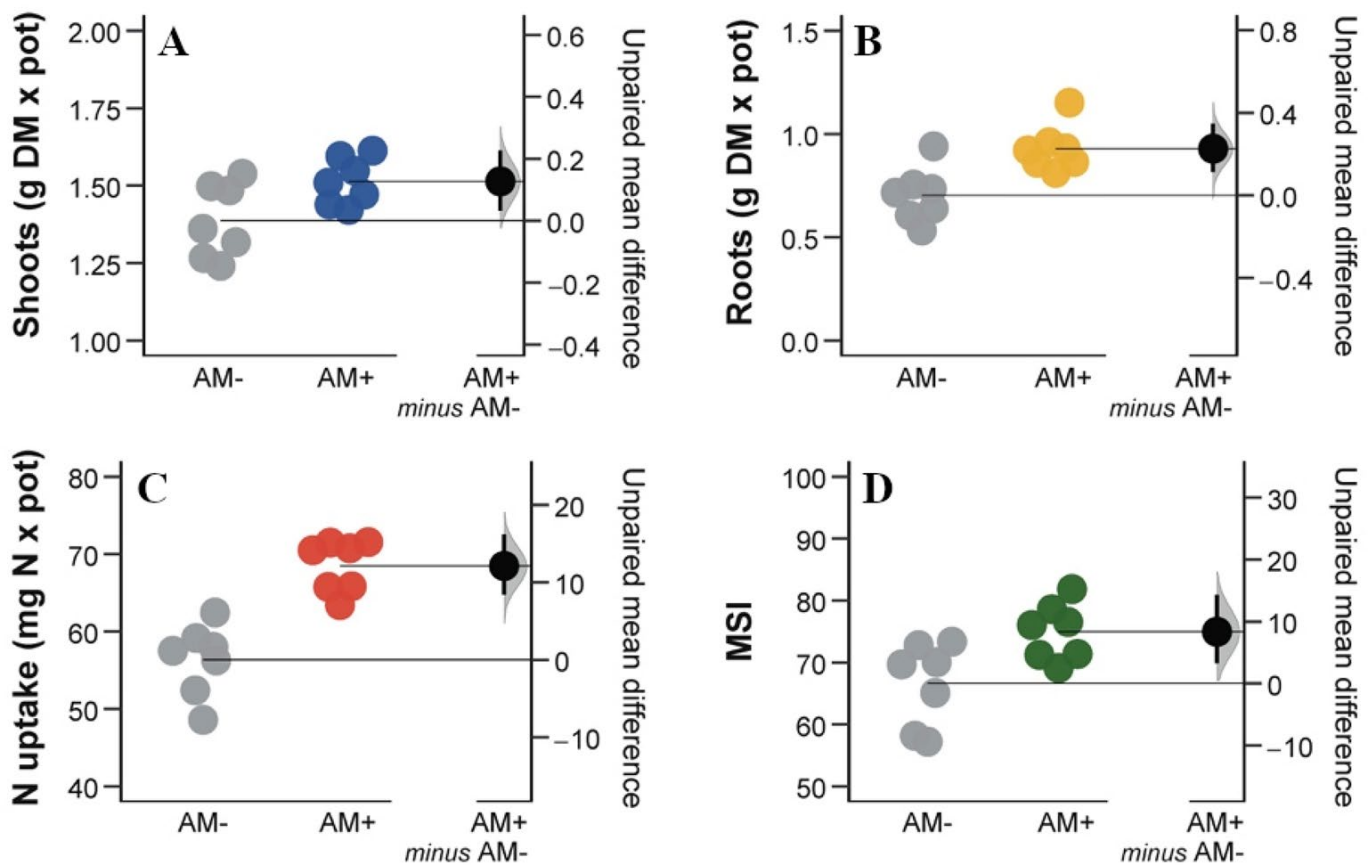
Shoot (**A**) and root (**B**) biomass, total N uptake (**C**) and membrane stability index (MSI; **D**). Raw data of Control (AM−, grey dots) and arbuscular mycorrhizal treatment (AM+, coloured dots) are shown in the plot. The filled curve indicates the resampled distribution of unpaired mean difference for AM + minus AM−, given the observed data. Horizontally aligned with the mean of the test group (AM +), unpaired mean difference is indicated by the black circle. The 95% confidence intervals of each difference is illustrated by the black vertical lines.

The mycorrhizal colonization of uninoculated plants was very negligible (on average 0.5% of the root length colonized with values always < 1%), whereas it was, on average, 31.2% (p value < 0.0001) in inoculated plants. AM + plants showed significantly higher aboveground and root biomass and total N uptake compared to the uninoculated (AM−) plants (+ 9.2%, + 32.1%, and 21.5% respectively; Fig. 1). Moreover, AM + plants had a higher membrane stability index (MSI) value compared to the AM− plants (+ 12.5% on average).

### Differential gene expression analysis

A RNA-Seq analysis on both Arbuscular Mycorrhizal fungi inoculated (AM +) and uninoculated durum wheat plants (AM−) grown under salt stress was performed to identify key pathways involved in the AMF-induced salt tolerance.

**Figure 2:**
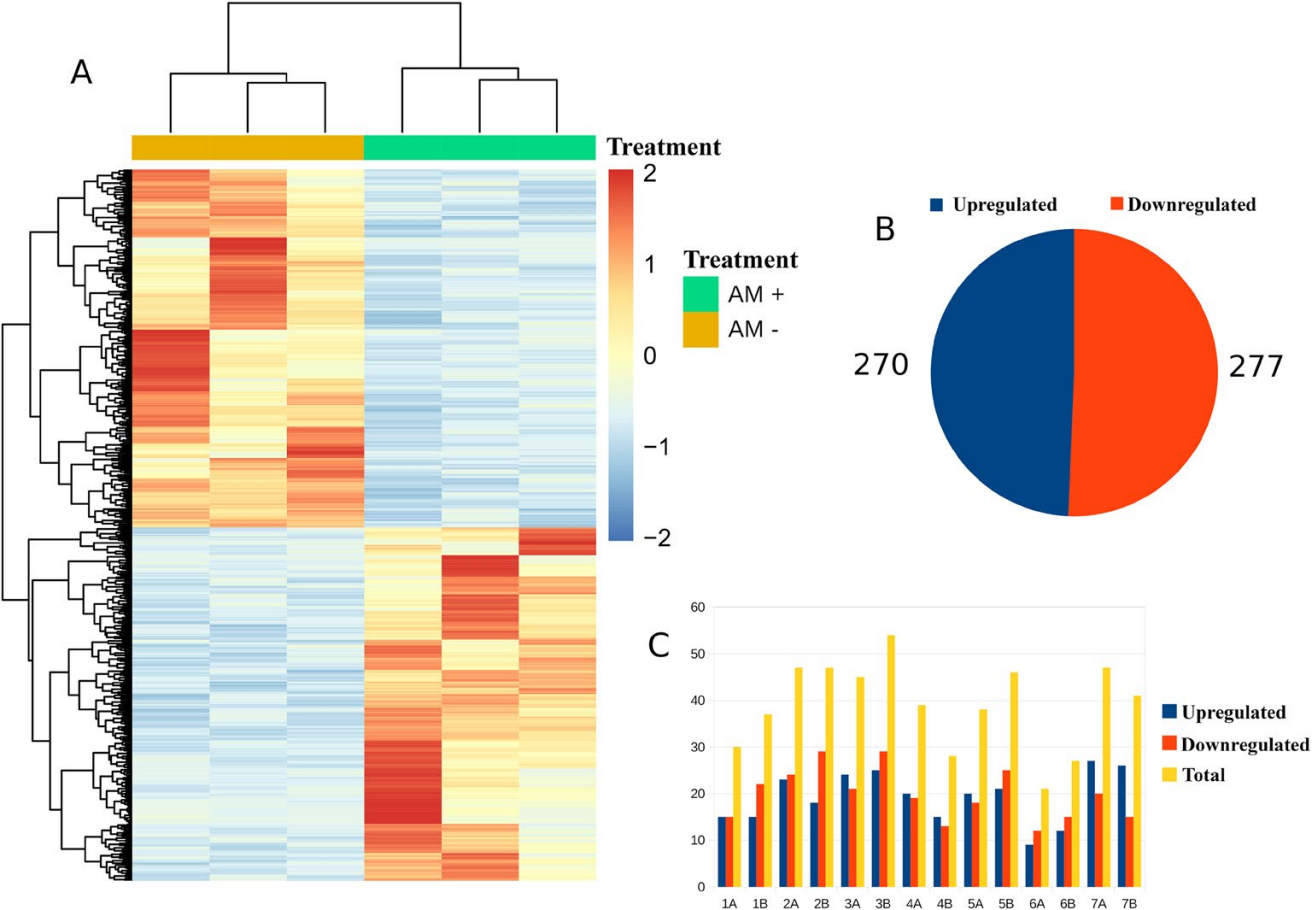
Overview of differential expression analysis performed with DESeq2. Heatmap of the normalized expression levels of the 563 Differentially Expressed Genes (DEGs) between the AM + and AM− conditions generated using the pheatmap R package (https://cran.r-project.org/web/packages/pheatmap/index.html; version 1.0.12) (**A**). Number of Differentially expressed genes in the two conditions (**B**). Distribution of DEGs in the 7 chromosomes of the *Triticum durum* genome (**C**).

Three hundred eleven (311) million single-end (SE) reads were obtained, with an average of 51 million reads per sample. Two hundred and seventy-eight (278) million reads were mapped to the durum wheat genome with an average of 77% mapping reads per sample. Five hundred and sixty-three (563) Differentially Expressed Genes (DEGs) between AM + and AM− samples were found, of which 277 with a log_2_FC < −1 and 270 with a log_2_FC > 1 (down- and upregulated by the AMF colonization, respectively) (Fig. 2A,B, Table S1). DEGs were distributed in all the seven chromosomes of both sub-genomes A and B. Their chromosome abundance was correlated with chromosome length (Pearson correlation, r = 0.83), and significant differences were not found between the sub-genomes (267 and 280 DEGs located on sub-genome A and B, respectively). The highest and lowest number of DEGs were found on chromosomes 3B (54) and 6A (21), respectively (Fig. 2C).

**Figure 3:**
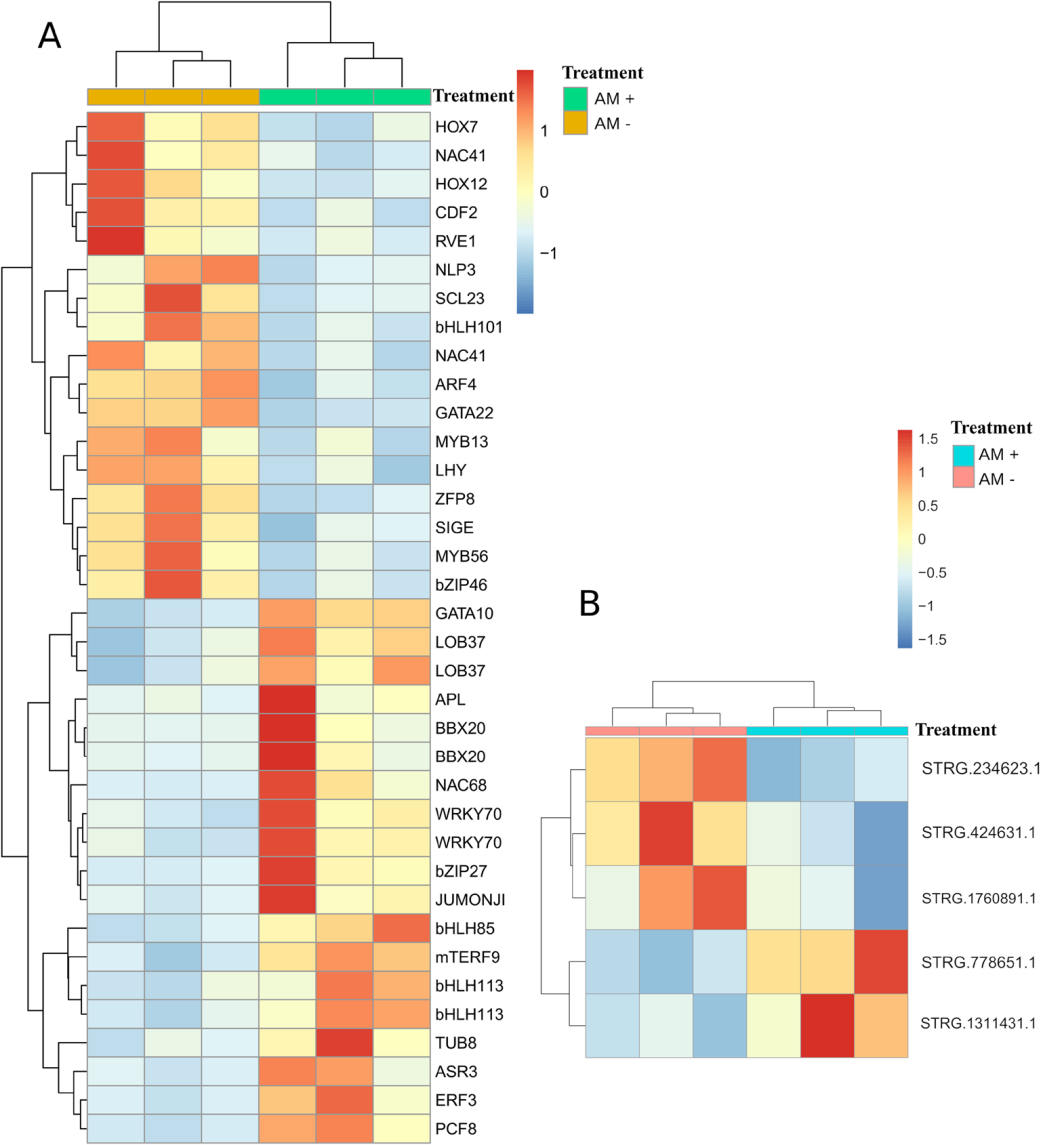
Expression patterns and hierarchical clustering of the 36 Transcription Factors identified comparing the AM + (green) and AM− (yellow) conditions (**A**). Heatmap showing the lncRNA differentially expressed under salt stress in the AM + (blue) and AM− (pink) conditions (**B**). Both heatmaps were generated using the pheatmap R package (https://cran.r-project.org/web/packages/pheatmap/index.html; version 1.0.12).

By using the durum wheat genome ncRNA annotation, lncRNAs were also investigated. Five lncRNA Differentially Expressed (DE) between treatments (Fig. 3B), two upregulated (STRG.778651, STRG.1311431) and three downregulated (STRG.234623, STRG.424631, STRG.1760891) by the AMF colonization were found. Interestingly, all the lncRNA DE were located on the sub-genome B in chromosomes 1, 2, 3, 5, and 7 and annotated using the RNAcentral sequence search tool (https://rnacentral.org/sequence-search/) (Table S2).

### Transcriptomic profile modification induced by AMF symbiosis

DEGs analysis between treatments revealed many genes involved in salt stress-related pathways such as ion transport and binding, response to oxidative and abiotic stress, calcium signaling, and osmolytes accumulation. AMF symbiosis significantly increased the expression levels of many genes previously reported as related to AMF-induced salt stress tolerance^12^ (Table 1). Concurrently a significant decrease in the expression of genes involved in the response to oxidative stress, GABA metabolism as well as ions and oligopeptides transport was observed. In particular, in the AM + samples, two catalase (CAT), two Glutathione S-transferase (GST), and twelve Cytochrome P450 (CYP) genes, all belonging to the enzymatic antioxidative systems, resulted downregulated. Furthermore, seven peroxidases (POX), also involved in the response to oxidative stress, were found DE (four upregulated and three downregulated).

**Table 1:** Key genes involved in the response to salt stress found differentially expressed between AM + and AM− samples. Log_2_ Fold Change (Log_2_FC), P-value, and Deseq2 adjusted P-value (Padj) are shown.

	Triticum durum gene ID	Log_2_FC	P-value	Padj	Transcript description
Oxidoreduction	TRITD6Av1G007920	− 2.2	3.9E−09	4.4E−06	Catalase
	TRITD6Bv1G012280	− 2.1	9.3E−08	6.2E−05	Catalase
	TRITD3Av1G174080	− 1.2	4.6E−05	7.4E−03	Glutathione S-transferase
	TRITD3Av1G263790	− 1.7	6.2E−06	1.7E−03	Glutathione S-transferase
Osmoregulation	TRITD3Av1G171110	− 1.7	1.1E−04	1.4E−02	Bidirectional sugar transporter SWEET
	TRITD3Bv1G149640	− 1.6	7.7E−04	4.9E−02	Bidirectional sugar transporter SWEET
	TRITD7Bv1G019190	− 2.1	4.0E−04	3.2E−02	Bidirectional sugar transporter SWEET
	TRITD1Bv1G219480	− 2.1	1.3E−06	5.4E−04	Glucose-1-phosphate adenylyltransferase
	TRITD1Av1G222050	− 2.2	2.0E−05	3.9E−03	Glucose-1-phosphate adenylyltransferase
	TRITD7Av1G204550	− 2.8	3.9E−05	6.7E−03	Polyamine oxidase 1
	TRITD7Bv1G158080	− 3.7	8.3E−09	8.2E−06	Polyamine oxidase
Transporters	TRITD4Bv1G194950	2.2	1.3E−05	2.8E−03	Aquaporin–like protein (TIP)
	TRITD3Av1G042320	− 1.3	2.9E−04	2.6E−02	ABC transporter
	TRITD5Av1G221130	− 1.5	4.2E−04	3.3E−02	ABC transporter
	TRITD6Bv1G179870	− 1.3	1.5E−05	3.3E−03	Auxin efflux carrier component (PIN)
	TRITD5Bv1G152290	− 2.5	1.3E−05	2.8E−03	Auxin efflux carrier family protein (PILS)
	TRITD5Av1G044900	1.4	1.0E−04	1.3E−02	Boron transporter
	TRITD5Bv1G042780	1.9	2.5E−04	2.3E−02	Boron transporter
	TRITD2Av1G215440	1.4	6.5E−04	4.4E−02	Mitochondrial Ca2 + Uptake protein
	TRITD6Av1G057110	1	6.7E−04	4.5E−02	Calcium-transporting ATPase
	TRITD6Bv1G067420	1	4.7E−04	3.5E−02	Calcium-transporting ATPase
	TRITD2Av1G032000	− 2.2	8.7E−07	3.9E−04	Cation calcium exchanger
	TRITD2Bv1G041270	− 1.7	5.2E−05	7.9E−03	Cation calcium exchanger
	TRITD7Av1G226260	− 1.2	4.5E−05	7.4E−03	HKT23 transporter
	TRITD7Bv1G172350	− 1	6.8E−04	4.5E−02	HKT23 transporter
	TRITD1Bv1G205440	− 1.7	2.1E−06	7.7E−04	Oligopeptide transporter
	TRITD1Bv1G205480	− 1.6	9.3E−05	1.2E−02	Oligopeptide transporter
NPF–NRT2	TRITD2Av1G001860	− 1.7	6.2E−05	9.0E−03	NPF transporter 8
	TRITD4Bv1G191460	− 1.4	1.8E−05	3.7E−03	NPF transporter 5
	TRITD5Av1G243130	− 1.8	1.7E−09	2.0E−06	NPF transporter 5
	TRITD6Bv1G164770	2.8	3.1E−04	2.7E−02	NPF transporter 7
	TRITD2Bv1G262700	1.1	1.0E−04	1.3E−02	NPF transporter 5
	TRITD7Bv1G180680	− 1.9	1.6E−04	1.7E−02	NRT2.2
Glutamate metabolic pathway	TRITD2Av1G208370	1.6	6.6E−04	4.5E−02	Glutamate decarboxylase
	TRITD4Bv1G016030	0.9	6.2E−04	4.3E−02	Glutamate decarboxylase
	TRITD7Bv1G022320	− 7.4	1.2E−07	7.4E−05	Glutamate receptor
Trehalose metabolic pathway	TRITD5Bv1G123710	2.8	7.0E−06	1.9E−03	Trehalose-6-phosphate synthase
	TRITD6Av1G158120	1.2	2.4E−04	2.3E−02	Trehalose 6-phosphate phosphatase
	TRITD6Bv1G144550	1.1	1.4E−04	1.5E−02	Trehalose 6-phosphate phosphatase
MAP Kinases	TRITD2Av1G270510	2	7.8E−06	2.0E−03	Protein kinase (MAP3K)
	TRITD6Bv1G202800	2.9	4.2E−06	1.3E−03	Protein kinase (MAPKK)
	TRITD2Av1G187690	1.2	5.5E−04	3.9E−02	Protein kinase (MAP3K)
Calcium signaling	TRITD5Bv1G079480	1.2	2.1E−04	2.1E−02	Calmodulin binding protein
	TRITD2Av1G044340	− 1.6	3.4E−06	1.1E−03	CIPK21
	TRITD2Bv1G037460	− 1.4	2.8E−07	1.4E−04	CIPK2
	TRITD5Bv1G024270	− 2.8	1.5E−05	3.2E−03	CIPK4
Other	TRITD3Av1G219080	2.2	2.2E−04	2.2E−02	Early response to dehydration 15-like
	TRITD3Bv1G211690	2.2	1.9E−04	2.0E−02	Early response to dehydration 15-like
	TRITD7Bv1G038640	1.3	4.6E−04	3.5E−02	Late embryogenesis abundant protein
	TRITD4Bv1G187750	1.5	8.4E−05	1.1E−02	Late embryogenesis abundant protein
	TRITD4Bv1G187770	2.4	4.5E−10	7.7E−07	Late embryogenesis abundant protein
	TRITD7Av1G099670	1.4	6.2E−05	9.0E−03	RAB GTPase
	TRITD3Bv1G206370	1.3	5.1E−04	3.7E−02	RAB GTPase

Interestingly, genes involved in osmoregulation and osmolytes accumulation such as sugars, amino acids, and trehalose were also modulated by AMF. In AM + plants, the expression of three sugar efflux transporters (SWEET) and eight invertases (beta-fructofuranosidase), involved in the conversion of sucrose to fructose and glucose, two ADP-glucose pyrophosphorylases and two polyamine oxidases (PAO) was significantly reduced. Furthermore, AMF colonization significantly enhanced the expression of a trehalose-6-P synthase (TPS) and two trehalose-6-P phosphatases (TPP), both responsible of trehalose biosynthesis, by contrast the trehalose degrading enzyme, trehalase (TRE), did not appear differentially expressed between treatments. Modulation of ion transporters genes is a key process for regulating the accumulation and compartmentalization of both ions and solutes. Four amino acid transporters (ANT and LHT) (two up- and two down-regulated) and twelve lipid-transfer proteins (eight up- and four down-regulated) were found differentially expressed. AMF symbiosis also enhanced the expression of a Tonoplast Intrinsic Protein (TIP) aquaporin, as well as ten transporter genes, including two borate transporters (BOR). By contrast, twenty-four transporter genes were significantly downregulated by the AMF colonization. Many of these are involved in amino acids, oligopeptides, and sodium transport such as three anion transporters, two auxin transporters, (PIN, PILS), two sodium HKT, two ABC transporters, and two oligopeptide transporters (OPT).

Among the nitrate transporters, five NPF and a NRT2 gene involved in both low- and high-affinity nitrate transport were differentially expressed between treatments. In detail, *NRT2.2* and three *NPF5* were down-regulated in AM + samples while a NPF7 and a NPF8 genes resulted upregulated.

In AM + plants, several genes involved in calcium signaling, such as calcium dependent transporters like Ca^2+^/cation exchanger (CCX) and Mitochondrial Ca^2+^ Uptake proteins (MICU), as well as three calcium-dependent protein kinases, *CIPK2*, *CIPK4*, and *CIPK21*, were found downregulated, while two P2B Ca^2+^ ATPase (*ACA*) and a calmodulin binding protein were upregulated. Furthermore, three genes involved in GABA metabolism, a GABA transaminase (*GABA-T*) and two glutamate decarboxylases (GAD), involved in GABA synthesis from glutamic acid, were also downregulated. Finally, in AM + plants, RAB GTPases, Late Embryogenesis Abundant (LEA) proteins, proline-rich proteins (PRP), and two MAP kinases (*MAP2K*, *MAP3K*) were significantly upregulated.

### Transcription Factors modulated by AMF symbiosis

The characterization of the expression profiles of Transcription Factors (TFs) is a crucial step in RNA-Seq analyses; here, thirty-six TFs were differentially expressed between treatments, of which 17 and 19 up- and down-regulated, respectively in AM + plants (Fig. 3A, Table S3). Many upregulated TFs were often involved in the response to abiotic and drought stress such as *WRKY*, Dehydration Responsive Element Binding (*DREB*), Early Response to Dehydration 15 like (*ERD15*), and *bHLH*. Among the downregulated TFs, two MYB genes (*MYB13* and *MYB56*), two *NAC*, and an Auxin Response Factor (*ARF4*) were identified.

### Profile validation of DEGs via qPCR

RNA-seq results were validated by qPCR using five randomly selected genes, two up- (*LEA* and *NRT1*), and three down-regulated (*HKT*, *MYB*, and *NRT2.2*). Their expression profiles were normalized to the housekeeping gene (Table S5). The expression trend of selected genes in qPCR (Fig. S2) appeared in agreement with the RNA-Seq expression levels. Pearson’s correlation coefficients were evaluated between RNA-seq and qPCR results showed high correlation and significance (R = 0.89; p < 0.0001).

### Gene Ontology (GO) and MapMan analysis

GO terms for Biological Process (BP), Molecular Function (MF) and Cellular Component (CC), were assigned to all the 563 DEGs and the enrichment analysis for both upregulated and downregulated genes was performed by using PANTHER.

**Figure 4:**
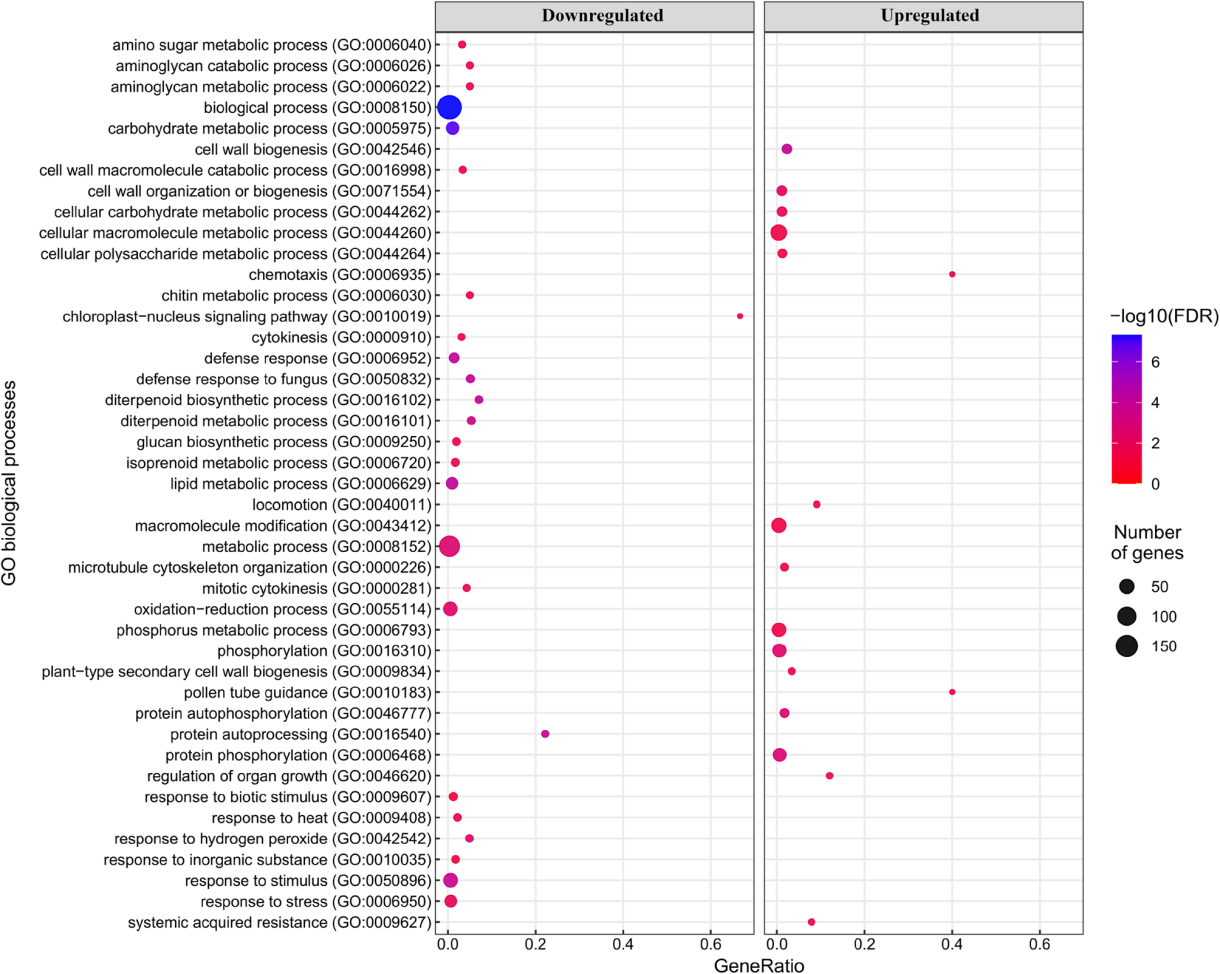
Biological Processes GO terms enrichment analysis for upregulated and downregulated genes. The dot plot shows enriched GO terms (FDR < 0.05) identified with PANTHER using the Fisher’s exact test. The size of the dots represents the number of genes in each GO biological process while the GeneRatio (x-axis) is the ratio between the number of DEGs found and the number of genes in that category.

In the BP category, enriched GO terms were not shared between the two sets of DEGs (up- and down-regulated by AMF inoculation). Many terms highly correlated to abiotic stress response such as “response to stress”, “response to stimulus”, “response to hydrogen peroxide”, “oxidation–reduction process”, “cell wall macromolecule catabolic process” and “lipid metabolic process” were found enriched in the downregulated genes set (Fig. 4). By contrast, the upregulated genes were mainly enriched in the GO terms “macromolecule modification”, “protein phosphorylation”, “protein autoprocessing”, “phosphorus metabolic process”, “cellular macromolecule metabolic process” and “cell wall biogenesis”.

In the MF category, “molecular function” and “catalytic activity” terms were shared between up- and down-regulated genes by AMF (Fig. S3). In addition, the downregulated genes were enriched in “hydrolase activity” and “oxidoreductase activity” terms, while three “purine binding” related terms, together with “ion binding”, “anion binding” and “carbohydrate derivative binding” terms were found enriched in the upregulated genes.

In the CC category, the “protein–containing complex” term was enriched in both up- and down-regulated sets of DEGs being also the only term enriched in the downregulated set (Fig. S4). Finally, other six terms were enriched in the upregulated genes,among which “cell wall”, “cell periphery” and “external encapsulating structure” appeared the most representative.

MapMan analysis highlighted the main metabolic pathways and functional groups involved in the response to salt stress comparing AM + and AM− treatments (Fig. S5). Three hundred eighty-nine out of 563 DEGs were correctly assigned to already known metabolic pathways. In detail, downregulated genes by AMF symbiosis were mainly involved in the amino acids biosynthesis and degradation, lipid metabolism, redox homeostasis process, terpenoids biosynthesis, phytohormone transport and biosynthesis as well as carbohydrate metabolism. By contrast, genes induced by AMF inoculation were mainly involved in RNA processing, vesicle trafficking, protein biosynthesis and cytoskeleton organization. Genes involved in solute transport, cell wall organization, RNA biosynthesis and external stimuli response were found in both up- and down-regulated genes sets.

### Co-expression network analysis

**Figure 5:**
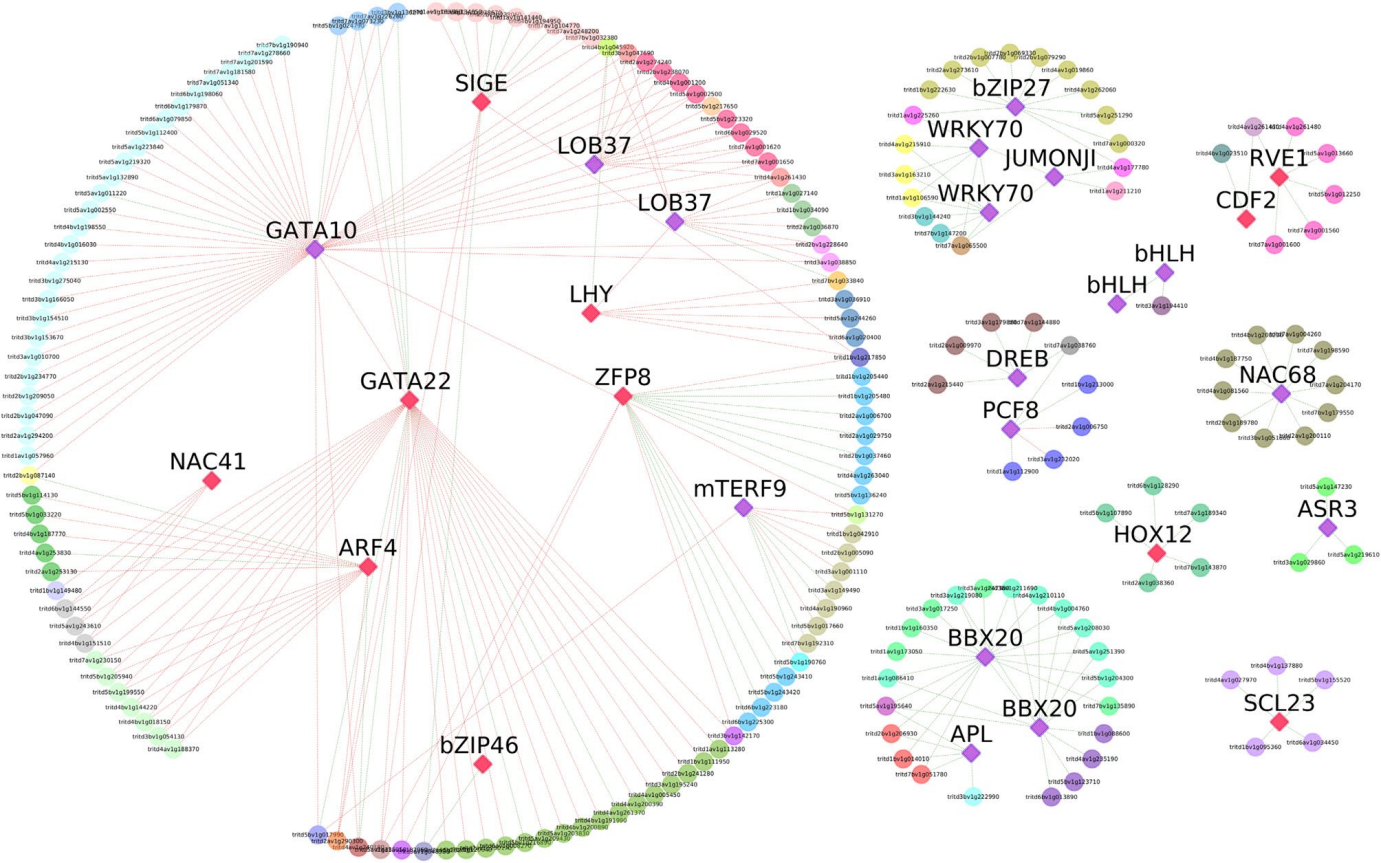
Co-expression network analyses of 30 Transcription Factors (TF) differentially expressed under salt stress as bait genes. The network was obtained with CoExpNetViz tool and visualized by Cytoscape. Bait TFs are represented as squared nodes. Colors represents downregulated TFs (red) and upregulated TFs (violet) while green and red lines denote positive correlation and negative correlation, respectively. Nine sub-networks are present, with the bigger one including 122 genes and 11 TFs.

The differentially expressed TFs panel to perform a co-expression network analysis highlighting specific interactions between key TFs and putative target genes was used. Only the TFs with a sub-network of at least two genes were considered for further analysis (six out of 36 were discarded). The network contains 230 co-expressed genes including 28 TFs that define a main sub-network of 122 genes and 11 TFs (4 and 7 up- and downregulated, respectively) and 9 smaller sub-networks (Fig. 5, Table S4). The main sub-network showed negative correlation between several TFs and their co-expressed genes, suggesting a TF repressive regulation. *zFP8* and *GATA10* TFs showed the highest number of connections inside the main network (hub genes). More interestingly, many DEGs previously mentioned for their important role in the salt stress response were found co-expressed together with some TFs utilized as baits. A HKT and PIN auxin transporter and a TPP gene resulted co-expressed to both GATA TFs. aBoth *BBX20* like TFs were connected to the up-regulated ERDs and a TPP gene, while *zFP8* with many OPTs, 5 Thaumatin-like proteins, and the *CIPK2* gene. Finally, a F-box (TRITD1Av1G225260) and a RING/U-box (TRITD4Av1G177780) genes, both involved in the ubiquitination process, were co-expressed with *bZIP27*.

## Discussion

Soil salinity is a relevant abiotic stress that can drastically limit crop growth and yield. High salt concentration in the soil is known to induce osmotic and oxidative stress and nutrient deficiency. Indeed, salinity can alter photosynthesis rates, cellular osmotic and ionic homeostasis through redox imbalances and accumulation of toxic elements (i.e., Na^+^ and Cl^–^), and can inhibit protein synthesis and deplete cellular energy.

Our findings showed that AMF symbiosis can mitigate the adverse effects of salt stress on durum wheat growth. In particular, we found that AM symbiosis favored N acquisition, and above- and below-ground plant growth under salt stress condition, in agreement with previous studies, as reviewed by Evelin et al.^12^. Moreover, we detected a clear positive effect of AM symbiosis on the alleviation of the damaging effects of salinity on the stability of plasma membranes. Abiotic stresses primarily target the cell membrane, and it is generally believed that maintaining their integrity and stability under stress conditions is a major component of salt stress tolerance in plants. We, therefore, concluded that mycorrhized plants are more resistant to salinity stress than their non-mycorrhized counterparts.

Non-colonized wheat plants exposed to salt stress showed lower MSI values than colonized plants, partially due to the salt affecting the electrical potential of the plasma membrane. As a result of AMF symbiosis, we observed a beneficial effect on MSI, which reduced the need to activate plants salt stress response mechanisms. Thus, AM + plants appeared subjected to a lower level of salt stress compared to AM− plants.

Several studies underlined the ability of AMF symbiosis to enhance plant salt tolerance by genes transcription modulation both in roots and shoot under different experimental conditions^4,12,21^. The protective effect of AMF under stress such as salinity, drought, and heat were already reported in durum and bread wheat^34,37,38^. Here, we described for the first time the differential transcriptomic profile of durum wheat leaves under salt stress with and without AMF inoculation. RNA-seq data were useful to describe the molecular mechanisms induced by AMF colonization to mitigate the salt stress in durum wheat. The identified DEGs between treatments revealed novel insights into the role of AMF symbiosis in the plant response to salt stress.

Soil salinity induces ROS formation and their accumulation both in roots and shoot tissues resulting in oxidative damage of cellular components. Plants react to ROS formation either by an enzymatic and non-enzymatic antioxidative system^39^. The enzymatic system determines a higher superoxide dismutase (SOD), catalase (CAT), glutathione reductase, and S-transferases (GR, GST), as well as peroxidase (POX) accumulation, while the non-enzymatic system involves the synthesis of antioxidant molecules such as ascorbate, carotenoids, and glutathione to counteract ROS accumulation toxicity^20^. In the present study, a higher number of enzymes involved in redox reactions such as CATs, GSTs, and CYPs significantly downregulated in the leaves of AM + plants were found. Thus, the AMF symbiosis abilityto suppress Na's antagonistic effect, reducing ROS accumulations, which could result in lower cellular and thylakoidal membranes damage, was suggested. CATs are involved in the enzymatic response to ROS^40^, GSTs reduce oxidative stress by affecting glutathione pools^41,42^, while CYP genes are known to provide tolerance to salinity and other abiotic stresses by influencing ROS scavenging and ABA levels^43^. Both GABA and glutamate metabolism and catabolism were also reported to play a key role in the response to salt stress by controlling ROS accumulation and regulating redox balance^44,45^. Interestingly, in AM + plants, we detected a significant downregulation of two glutamate or Glutamic Acid Decarboxylase (GAD) enzymes involved in the synthesis of GABA from L-glutamate as well as a GABA-T involved in the conversion of GABA to succinic semialdehyde (SSA), already reported to be induced by salt stress in Arabidopsis and maize^46,47^.

Otherwise, N-compounds have been suggested to contribute to osmo-protective processes and to mitigate oxidative stress by scavenging ROS^23^. Here, three NPF transporters and a NRT2 transporter resulted downregulated by the AMF symbiosis. In agreement, a significant reduction in the expression of genes involved in the N uptake was induced by ectomycorrhizal fungi inoculation in poplar^28^; by contrast, tomato plants under both drought and salt stress showed a NRT2 genes^48^ upregulation^28,48^. In our condition, AM + plants exhibited an improved ability to uptake and translocate N, resulting in a reduced expression of high-affinity transporters in leaves probably due to a higher nitrate level in the xylem.

Two HKT genes involved in the Na^+^ transport from shoot to roots, thus able to remove Na^+^ from the xylem appeared significantly downregulated in the AM + samples, in contrast with previous findings in which the AMF symbiosis determined their upregulation^21^. *HKT*s down-regulation may suggest a higher ionic balance in the AM + plants in agreement with Estrada et al. ^49^ that measured a higher K^+^/Na^+^ ratio in AM + samples as well as a higher state of oxidative stress in AM− samples. This last condition may be caused by an inefficient water and ionic homeostasis, highlighted by enriched GO terms identified in the downregulated genes such as “oxidation–reduction process” and “response to stress”. The reduction of plant defense responses to salt stress in the oxidation–reduction reactions observed in the shoot of AM + compared to AM− plants is probably due to their healthier condition.

To mitigate the adverse effects of the low soil water potential caused by salinity, plants accumulate osmolytes such as proline, betaine, polyamines, sugars, organic acids, amino acids, and trehalose, aiding the water flow from the soil into the roots and then to shoots^12^. Higher osmotic potential in AM + compared to AM− plants has been already reported^50^. In our study, a high number of Proline-Rich Proteins (PRPs) upregulated by the AMF symbiosis were detected. These seem to be involved in cell wall stress-induced fortification as confirmed by six cell wall-related GO terms enriched in the upregulated genes, including “cell wall biogenesis” and “cell wall organization”. Indeed, proline is an important osmo-protectant, accumulated under salt stress as free proline or in PRPs, mainly in cell walls^51,52^. Trehalose is a non-reduced storage disaccharide that may serve as energy and carbon source, seeming also involved in both ROS scavenging and K^+^/Na^+^ ratio maintenance^53^. A higher levels of trehalose in legume grown under salt stress were reported^54^. In agreement, a trehalose-6-P synthase (TPS) and two trehalose-6-P phosphatases (TPP), involved in the trehalose synthesis, resulted strongly upregulated in AM + durum wheat plants.

Moreover, several genes involved in polyamines and carbohydrates metabolisms resulted downregulated by AMF inoculum, in agreement to the GO term “carbohydrate metabolic process”, significantly enriched in the downregulated genes set. Polyamines are important regulators of cellular ROS homeostasis^55^, while the accumulation of soluble carbohydrates was detected in the leaves of salt stressed sorghum plants^56^.

Changes in cytosolic free Ca^2+^ concentration are known to be involved in message transduction and response to several external stimuli, biotic and abiotic stresses^57,58^. During drought and salt stress, a high number of calcium-related genes involved in the Ca^2+^ signaling pathway are induced. In our comparison, a calmodulin-binding protein and two P2B Ca^2+^ATPase, both responsible for the active Ca^2+^ transport and homeostasis, were upregulated by the AMF symbiosis. More interestingly, these genes were already reported as involved in salt stress responses in moss, soybean, and rice plants^59–62^. Finally, three MAPKs, involved in different signaling cascades including Ca^2+^, resulted upregulated in the AM + plants.

In our condition, the AMF colonization downregulated three CIPKs and two Ca^2+^/cation exchangers (CCX). Interestingly, CIPKs are involved in Ca^2+^ signal transduction in abiotic stress responses in many crops^57,63,64^, by contrast, the role of CCXs is still scarcely characterized. They localize to the vacuolar or plasma membrane and could be involved in Na^+^/K^+^ exchange and stress response having been found highly upregulated during both salt and drought stress in many plants, despite limited knowledge on their regulation and specific functions is available until now^65–67^.

The aquaporins form a large protein family with a pivotal role in plant water use efficiency (WUE) and water transport. A complex transcriptional pattern of aquaporins under salt stress has been described in several crops^68–70^. Both Tonoplastic and Protoplastic Intrinsic Proteins (TIPs and PIPs), involved in the intracellular and transcellular water transport, respectively, under salt stress were found modulated by the AM fungi both in roots and leaves^71^. Here, we identified a TIP gene upregulated by the AMF inoculation.

Many transcription factors belonging to DREB, NAC, MYB, bHLH, bZIP, ERF, BBX, and WRKY families play a significant role in biotic and abiotic stress plant responses^72–75^. We identified seventeen TFs upregulated by the AMF symbiosis among which two *WRKY70* homologs, a *DREB*, a *NAC68* homolog, and two *bHLH*. The overexpression of WRKY, NAC, and DREB members were able to enhance abiotic and biotic stress responses in rice, *Arabidopsis* and *Nicotiana benthamiana*^76–79^. In wheat, the overexpression of both *NAC29* and *bZIP15* has been recently reported to enhance salt stress response by reducing H_2_O_2_ accumulation^80,81^. Furthermore, two *BBX20* TFs were significantly upregulated by the AMF symbiosis in our experiment, in agreement with the higher expression of this family members previously reported in several plants under salt and heat stress^82,83^.

In our condition, *MYB13* and *MYB56* were also identified among the downregulated TFs in AM + plants, and both members were reported to induce drought, salt, and cold stress tolerance in transgenic *Arabidopsis*^84,85^, suggesting a lower oxidative stress level.

Interestingly, RAB-A, LEA, and ERD genes were identified among the upregulated DEGs induced by AMF inoculation. RAB-A GTPases are involved in membrane trafficking, and signal transduction and appeared involved in salt stress tolerance^86,87^. Members belonging to the LEA gene family enhanced salt and dehydration stress tolerance when overexpressed in transgenic plants^88–90^. LEA proteins have been also reported to act as a molecular shield during abiotic stresses avoiding protein aggregation and preventing enzyme degradation^91^.

Finally, long noncoding RNAs (lncRNAs) have been found frequently induced during abiotic stress plant responses^92–94^. Here, we detected two lncRNAs upregulated and three down-regulated by the AMF symbiosis under salt stress. Interestingly, the ortholog of a drought-responsive lncRNA (URS0000781584) identified in maize by Zhang et al. resulted downregulated in our experiment^95^.

In conclusion, we observed a lower number of stress-related genes modulated by soil salinity in AM + compared to AM− samples. This appears in agreement with the results previously obtained on bread and durum wheat^26,34^, supporting the hypothesis of a salt stress mitigation induced by AMF symbiosis. The DEGs identified in our condition included plant defensive genes against redox state (i.e., cytochrome P450, glutathione S-transferase, catalases) and were involved in osmoregulation, osmolytes, and ions transport. These observations were confirmed by the GO-term enrichment analysis that showed categories related to stresses (i.e., “response to stress”, “response to hydrogen peroxide”, and “oxidation–reduction process”) significantly enriched in AM- plants. More interestingly, MapMan analysis identified a higher number of genes involved in pathways related to abiotic stress responses such as amino acids and carbohydrate biosynthesis and metabolism (involved in osmotic regulation), lipid metabolism (dehydration protection), redox homeostasis process (anti-radical responses), terpenoids biosynthesis (antioxidant protection) and phytohormone transport (stress response modulation). Finally, the co-expression network analysis was able to identify many TFs acting as hub-genes in the regulation of the higher salt tolerance mediated by AMF-inoculum in durum wheat.

Altogether, our molecular findings well correlate with a higher salt stress tolerance previouslyobserved in AM + plants and may be considered a gene atlas that clearly sustains a healthier status of AMF colonized compared to non-colonized plants. Further studies will be necessary to narrow down the genes set here identified to isolate candidate major genes involved in AMF-mediated salt tolerance for further functional analyses. The adoption of different AMF species, salt concentrations, and times of exposure to stress may help to improve our knowledge on the interaction between plants and AMF.

## Methods

### Plant material and experimental design

Durum wheat (*Triticum durum* Desf. cv. Anco Marzio) plants were grown outdoors in pots under salt stress with and without AMF inoculation (namely ‘AM+’ and ‘AM−’, respectively). A complete randomized design was adopted with seven replicates (for a total of 14 pots). Each pot (diameter 150 mm, height 130 mm) was filled with 2000 g of a quartz sand:soil mixture (1:1). Soil properties were as follows: 267 g kg^–1^ clay, 247 g kg^–1^ silt, and 486 g kg^–1^ sand; pH 8.0; 6.3 g kg^–1^ total carbon (C); 0.86 g kg^–1^ total N; available P (Olsen) 40.1 mg kg^−1^; 1.70 dS m^–1^ saturated electrical conductivity (EC) (25 °C). Both soil and sand were sieved through a 2 mm mesh and autoclaved at 121 °C for 20 min to completely impair soil biological (both fungal and bacterial) activity. The native bacterial microflora was extracted by suspending 500 g of fresh soil in 1.5 l distilled water. After shaking and decanting, the suspension was filtered (11 μm mesh) to discard the native AMF community. Each pot received 30 ml of soil suspension filtrate to reintroduce the native microbial community before AMF inoculation, performed with 10 g per pot rate of a commercial AMF inoculum (AEGIS IRRIGA, Italpollina SpA, Rivoli Veronese, Italy), consisting of a *Rhizophagus irregularis* and *Funneliformis mosseae* spore mixture, at 700 spores g^–1^ of inoculum rate for each. The commercial inoculum also contains 1 × 10^7^ rhizosphere bacteria. To isolate the AMF effects, we extracted the bacterial community belonging to the inoculum, using the same protocol applied for the native soil microbial community reported above, and introduced it to the AM- treatment. The microbial inoculations were performed at the same time as sowing. The native microbiome and the bacterial community present in the inoculum (only for AM− treatment) were added in liquid form as reported above, while AM fungal inoculum was distributed just below the sowing bed. Each pot received 60 mg of N in the form of ammonium sulfate ([NH_4_]_2_SO_4_).

Sixteen seeds, previously surface-sterilized with H_2_O_2_ at 4% for 3 min, were sown in each pot. Ten days after emergence, plants were thinned to six seedlings per pot. The plantlets were grown for 15 days before the application of salt to avoid its negative effect on the AMF symbiosis establishment. Salt stress was determined by adding NaCl to the irrigation water (10 g l^–1^). To prevent osmotic shock, salt was added gradually by distributing in total 1 l of NaCl solution in each pot within seven days from the beginning of the salinity treatment. This treatment led the EC of saturated soil extract to 13.00 dS m^–1^. Afterward, plants were watered with tap water (0.58 dS m^–1^) until harvest. Leaching was avoided by maintaining soil water always below field capacity. During the experiment, the irrigation was performed every 2 days and the amount of irrigation water consisted of the total replenishment of water lost through evapotranspiration for each pot.

All pots were harvested after 45 days from sowing. Plant aboveground biomass was immediately separated into stems, green leaves, and senescent and dry leaves, and the fresh weight of each fraction was recorded. Root biomass was extracted by carefully cutting the pots vertically and removing the substrate by washing. About 1 g of green leaves and 1 g of roots from each pot were immediately frozen in liquid N, stored at − 80 °C, and subsequently pulverized without thawing. At the same time, a sample of green full expanded leaves (about 400 mg) was taken from each pot to determine the membrane stability index (MSI). The leaf material was divided into two sets of 200 mg each. The first set was heated at 40 °C for 30 min in a water bath (10 cm^3^); then the electrical conductivity bridge (C1) was measured. The second set was boiled at 100 °C for 10 min (in 10 cm^3^ of water) before measuring the electrical conductivity bridge (C2). MSI was calculated according to Sairam et al.^96^.

A representative root sample (about 1 g) was taken from each pot to determine the overall colonization of roots by AM fungi. To this end, root samples were cleared in KOH and stained with trypan blue following the method described by Phillips et al.^97^. AM fungi root colonization was then measured with the grid intersect method^98^.

Plant N content was determined separately on dry material of each botanical fraction obtained as previously described using the combustion method of Dumas (DuMaster D-480, Büchi Labortechnik AG, Flawil, Switzerland). For each pot, total N uptake was calculated as the sum of N accumulated in roots (root dry mass × root N concentration) and shoots (shoot dry mass × shoot N concentration).

All plant data were compared between the two groups (AM + and AM−) using the dabestr R package^99^ to generate unpaired mean differences via a bias-corrected and accelerated bootstrapped 95% Confidence Intervals (Cis). Graphical data representations were generated using the dabestr R package. All analyses were performed using R version 4.0.2^100^.

### RNA-Seq library preparation and sequencing

Total RNA from leaves using the Spectrum Plant Total RNA kit (Sigma) and treated with RNase-free DNase was isolated. RNA quantification was analyzed by the Nanodrop^®^ND-1000 (Thermo Scientific,Walthman, MA, USA) and its quality (RNA integrity number—RIN > 8.0) was assessed using an Agilent Bioanalyzer RNA nanochip (Agilent, Wilmington, DE, USA). Three biological replicates for each treatment were used. Each replicate included a pool of healthy fully expanded leaves taken from all the plants in the pots. Sequence libraries were prepared as reported in Puccio et al.^101^ using a TruSeq RNA Sample Preparation Kit v2 (Illumina, San Diego, CA, USA). Both quality and insert size distribution by using an Agilent Bioanalyzer DNA 1000 chip were assessed. Sequence libraries were pooled in equimolar concentration and analyzed on an Illumina HiSeq 2000 generating 75 bp reads. The generated sequences were deposited in the NCBI (National Center for Biotechnology Information) SRA database with the accession: PRJNA746118.

### Reads pre-processing and mapping

The quality assessment of reads obtained from both AM inoculated (AM +) and non-inoculated (AM−) samples were performed using the FastQC tool v0.11.8^102^. Sequence trimming was not performed and the high sequencing quality with a median per base sequence quality of 36 (Phred Score) and the absence of adapter sequences was reported (Fig. S1). Reads were mapped to the newly released *Triticum turgidum* L. ssp.* durum* genome^103^ using STAR^104^ with default parameters and assigned to genomic features using featureCounts^105^ with default parameters.

### Differential expression analysis and annotation

Differentially expressed genes (DEGs) and long non-coding RNA (lncRNA) between treatments were obtained using the DESeq2 R package, using an adjusted p-value (padj) < 0.05 as threshold^106^. DEGs were annotated using the *Triticum durum* genome annotation, the *Triticum aestivum* orthologues from the biomart plants database,the online PANTHER functional classification^107^ and visualized using the pheatmap R package (version 1.0.12)^108^. DEGs were also assigned to specific metabolic pathways using the online functional annotator Mercator^109^ and visualized with MapMan^110^. Furthermore, differentially expressed (DE) lncRNA were annotated using the nhammer function of the online database RNAcentral^111^.

### GO enrichment and co-expression network analysis

GO enrichment analysis was performed with PANTHER overrepresentation test using the Fisher’s exact test and a False Discovery Rate (FDR) threshold of 0.05. Enriched GO terms were then filtered with the REViGO tool^112^ to reduce GO terms redundancy and visualized using the R package ggplot2^113^.

A Co-expression network analysis was performed by the CoExpNetViz tool^114^ using the 1st and 99th percentiles of the Pearson correlation coefficients distribution, as thresholds. Differentially expressed (DE) transcription factors (TFs) were used as bait genes while the entire set of DEGs was used as pool. The Network obtained was visualized and analyzed using Cytoscape^115^ using the network analyzer tool.

### RNA-Seq analysis validation using qPCR

To validate the results from the RNAseq analyses, a set of five genes were randomly selected among DEGs (Table S5) and tested by qPCR, using the actin gene as reference^116^. Primer 3.0 software (http://primer3.ut.ee/) (accessed on 16 October 2021) to design primer pair for each selected gene was utilized (Table S5). Total RNA from leaves of durum wheat plants belonging to an independent experiment performed following the same procedures described in “Plant material and experimental design” section was isolated. Reverse transcription was performed on 200 ng of total RNA extracted from AM− and AM + samples, using iScript Reverse Transcription Supermix (Bio-Rad, Berkeley, CA, USA), according to manufacturer’s instructions. qPCR was performed as described in Puccio et al.^101^, starting from 20 ng of cDNA^101^. Three biological and three technical replicates were analyzed for each sample (AM + and AM−). Fragment amplification was verified by 1.5% w/v agarose gel electrophoresis and melting curve analysis. The relative expression ratio of each gene was calculated by the 2^−ΔΔCT^ method^117^. Pearson correlation analysis between RNA-Seq and qPCR was also performed.

### Legislation statement

All procedures were conducted in accordance to the institutional, national, and international guidelines and legislation.

## Supplementary Information

Supplementary Figures.

Supplementary Tables.

## Data Availability

The datasets supporting the conclusions of this article are available in the Sequence Read Archive (SRA) repository (http://www.ncbi.nlm.nih.gov/sra/), accession number: PRJNA746118.
