# Supplementary material for: Transcriptome changes induced by Arbuscular mycorrhizal symbiosis in leaves of durum wheat (Triticum durum Desf.) promote higher salt tolerance
Source: Sci Rep. 2023 Jan 3;13:116. doi: 10.1038/s41598-022-26903-7 (PMC9810663; doi:10.1038/s41598-022-26903-7)

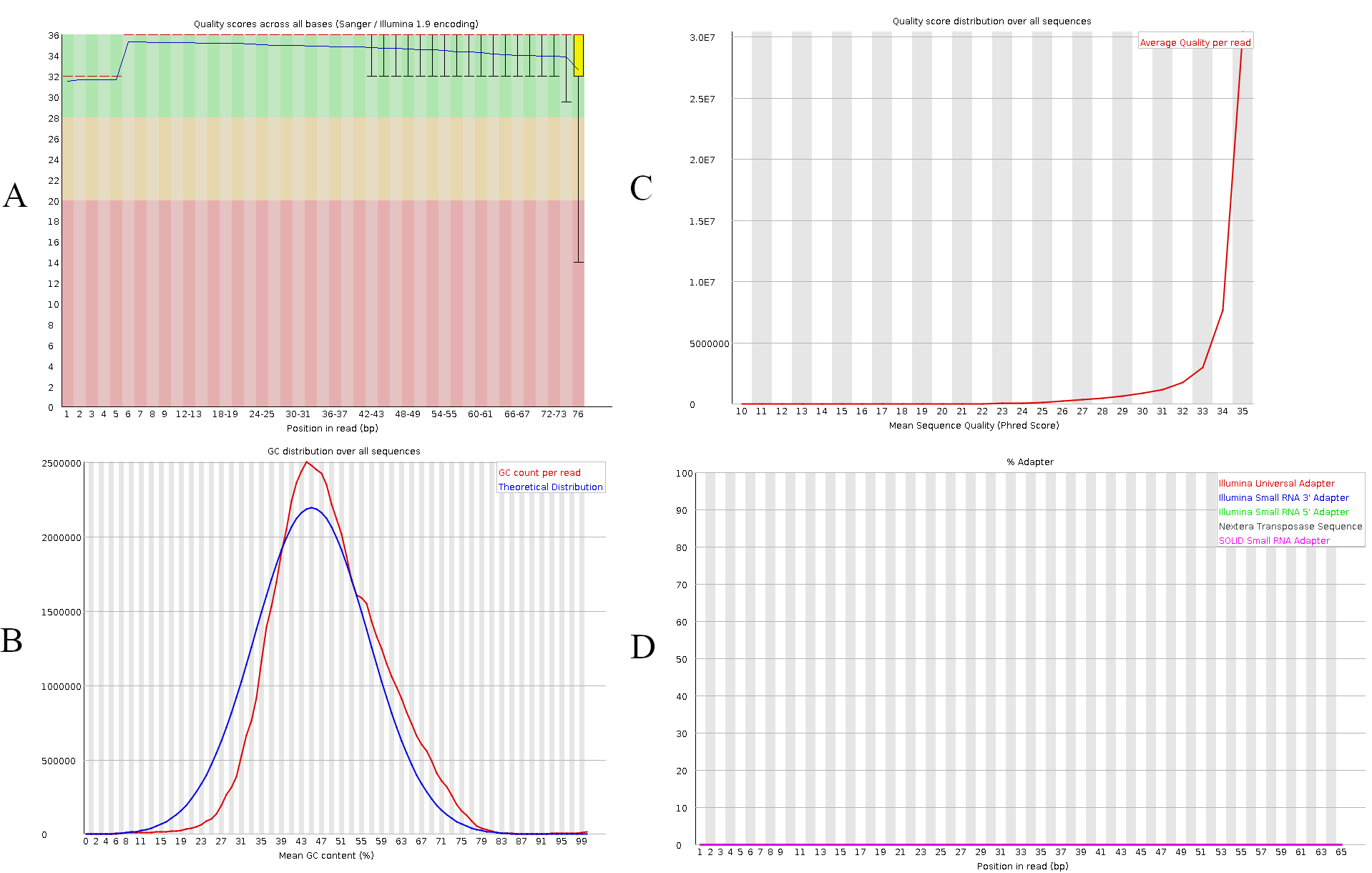
**Figure S1** Sequencing quality overview obtained using FastQC. **A** Per base sequence quality with quality scores on the y-axis and read positions on the x-axis. **B** GC percentage plot. Calculated GC content reference distribution is shown in blue while sequencing GC content distribution is shown in red. **C** Per sequence quality score distribution with Phred Score on the x-axis and the number of sequences on the y-axis. **D** Adapter sequence content plot.

**Figure S2** Expression levels of genes selected to validate RNAseq profiles
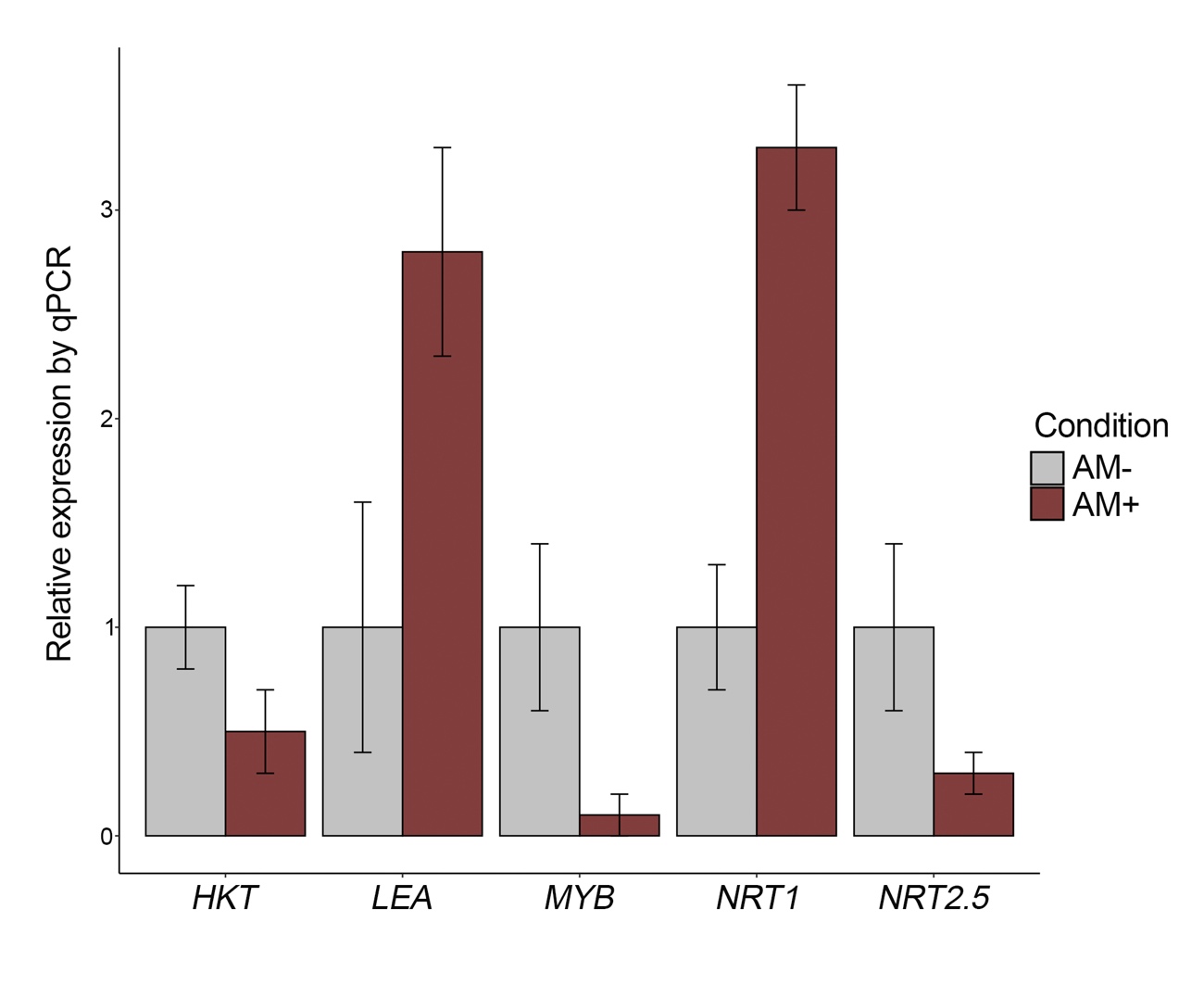


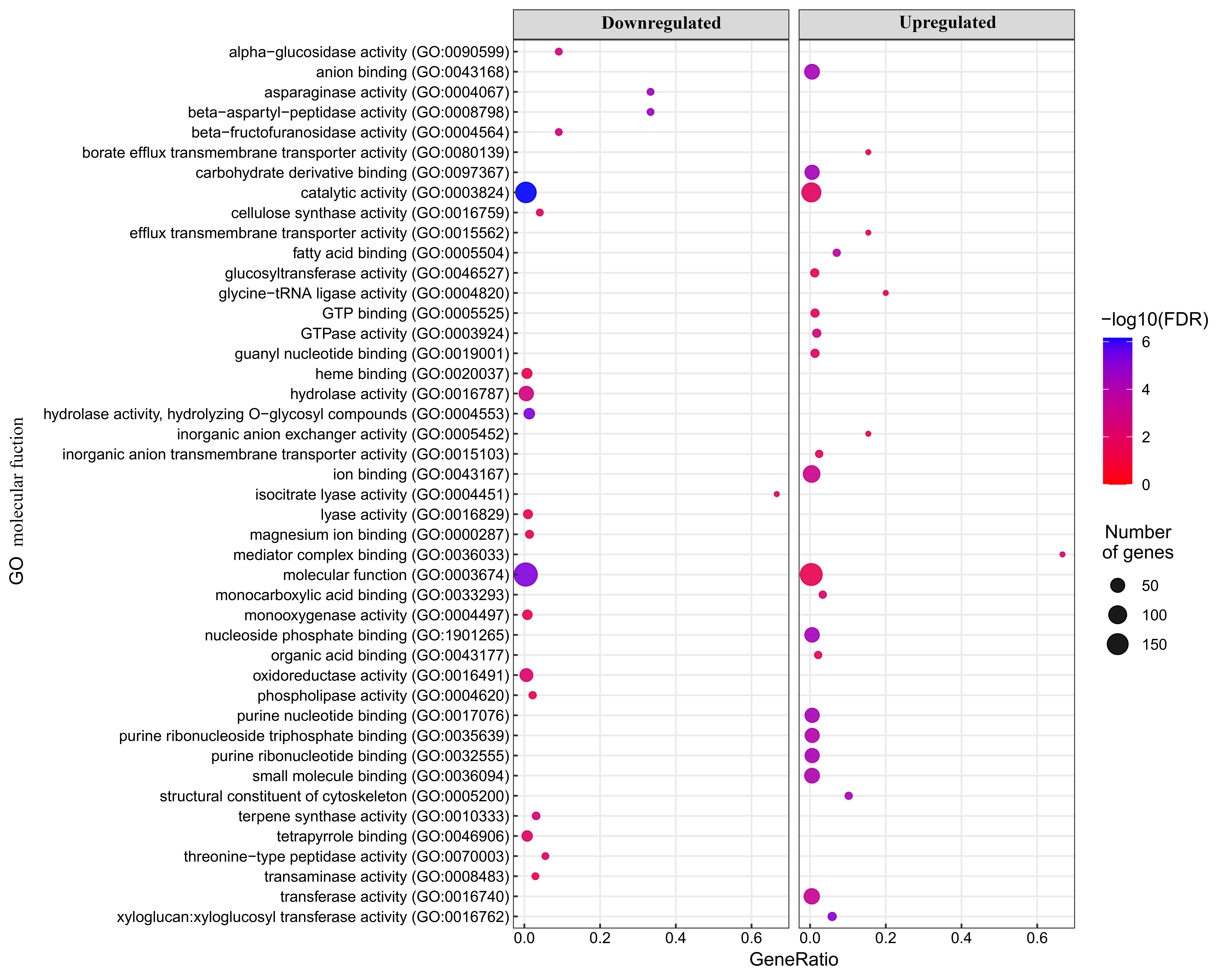
**Figure S3** Molecular function GO terms enrichment analysis for upregulated and downregulated genes. The dot plot shows enriched GO terms (FDR < 0.05) identified with PANTHER using the Fisher’s exact test. The size of the dots represents the number of genes in each GO molecular function while the GeneRatio (x-axis) is the ratio between the number of DEGs found and the number of genes in that category.

**Figure S4** Cellular component GO terms enrichment analysis for upregulated and downregulated genes. The dot plot shows enriched GO terms (FDR < 0.05) identified with PANTHER using the Fisher’s exact test. The size of the dots represents the number of genes in each GO cellular component while the GeneRatio (x-axis) is the ratio between the number of DEGs found and the number of genes in that category.
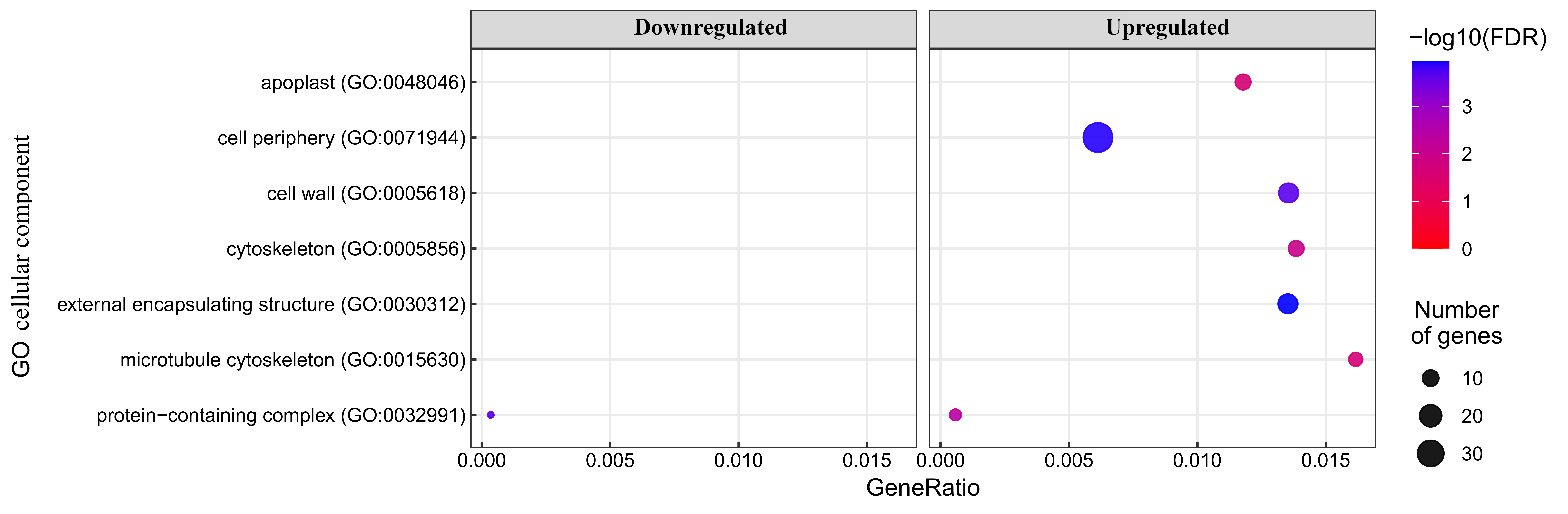


**Figure S5** MapMan analysis graphical output. Each square represents a DEG while the color represents the log2FC (> 1 for AM-; < -1 for AM+). Overview of MapMan BINs (**A**). Abiotic/Biotic stress response (**B**). Metabolism overview (**C**).
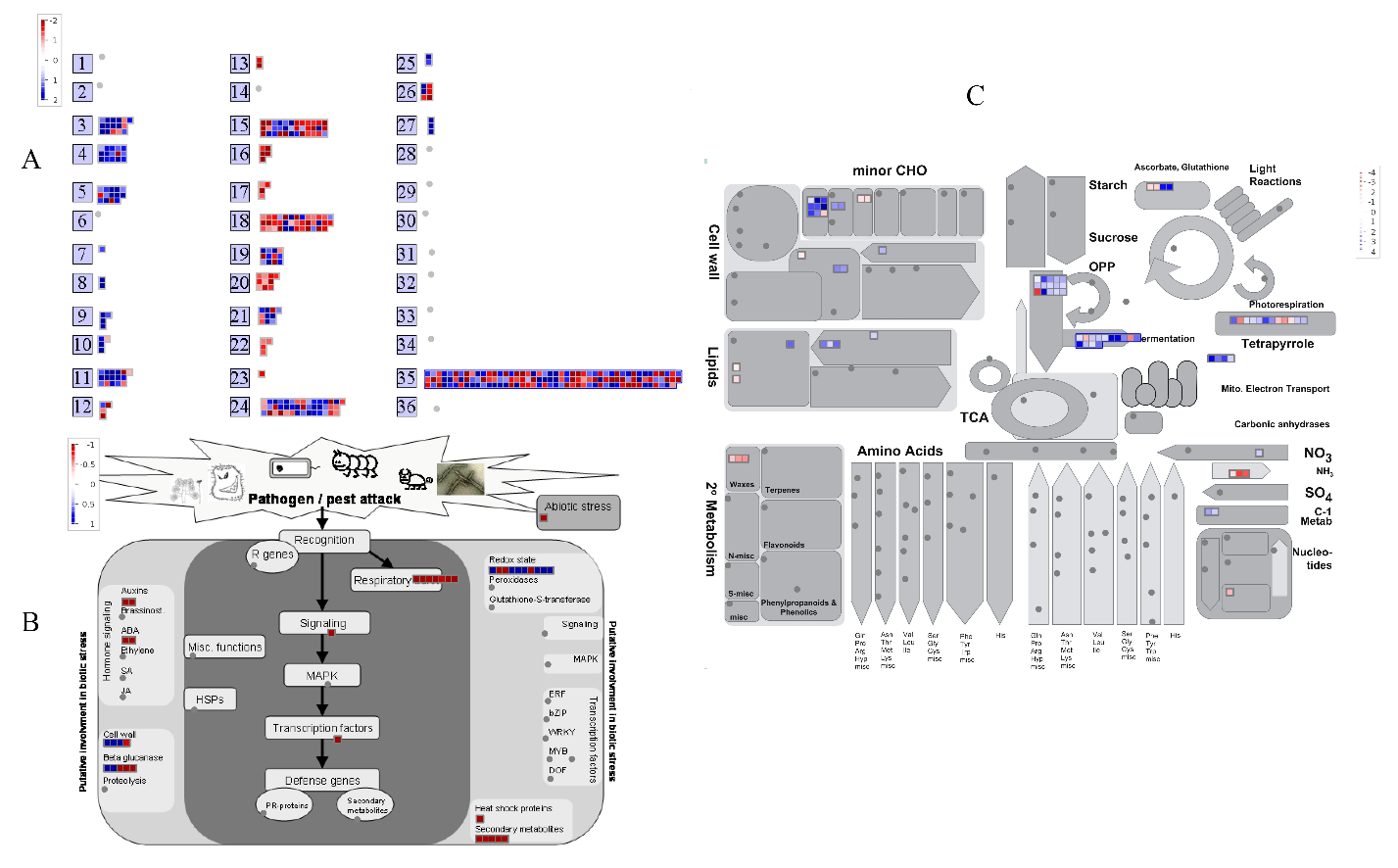

Supplement: Supplementary file 1 — Supplementary Figures. [file 41598_2022_26903_MOESM1_ESM.docx]
